# Over-expression of cathepsin B in hepatocellular carcinomas predicts poor prognosis of HCC patients

**DOI:** 10.1186/s12943-016-0503-9

**Published:** 2016-02-20

**Authors:** Jian Ruan, Haiyan Zheng, Xiaodong Rong, Xiaomin Rong, Junyi Zhang, Weijia Fang, Peng Zhao, Rongcheng Luo

**Affiliations:** Cancer Center, Traditional Chinese Medicine-Integrated Hospital, Southern medical University, Guangzhou, 510515 Guangdong Province People’s Republic of China; The Institute of Gynecology and Obstetrics, The Third Affiliated Hospital of Guangzhou Medical University, Guangzhou, 510315 Guangdong Province People’s Republic of China; Department of Radiation Oncology, Sun Yat-sen University Cancer Center, Sun Yat-Sen University, Guangzhou, 510000 Guangdong Province People’s Republic of China; Department of pharmacy, Sun Yat-Sen Memorial Hospital, Sun Yat-Sen University, Guangzhou, 510000 Guangdong Province People’s Republic of China; Department of Medical Oncology, The First Affiliated Hospital, School of Medicine, Zhejiang University, Hangzhou, 310003 Zhejiang Province People’s Republic of China

**Keywords:** Hepatocellular carcinoma, Cathepsin B, Prognostic marker

## Abstract

**Background:**

Several studies have found that Cathepsin B (CTSB) is up-regulated in many tumor types and facilitates tumor progression. However, the role of CTSB in hepatocellular carcinoma (HCC) progression remains unclear. This study was aimed at investigating the expression and role of CTSB in HCC in a large set of samples and cell lines (MHCC-97H and MHCC-97 L), and evaluating the clinical and prognostic significance of CTSB protein in patients with HCC.

**Methods:**

The expression of CTSB was examined in HCC tissue and cell lines by Western-blotting, Real-time PCR, and immunohistochemical staining. Wound healing assay and invasion assay were used to verify the effect of CTSB on the migration and invasion ability of HCC cell lines. Tumor formation assay in nude mice was used to analyze the effect of CTSB on the tumorigenicity of HCC cell lines.

**Results:**

The status of CTSB protein in carcinoma tissues is much higher than that in paracarcinoma tissues. The overall survival of the patients with high CTSB expression was significantly shorter than the low CTSB expression group. High CTSB expression was significantly correlated with advanced clinical staging, histological grade, and tumor recurrence. In vitro and in vivo experiments demonstrated that over-expression of CTSB in MHCC-97 L cells promoted cell invasion and tumor progression ability. Down-regulation of CTSB in MHCC-97H showed the opposite effects. These phenotypic changes caused by CTSB knockdown or over-expression correlated with expression of the matrix metallopeptidase MMP-9. Moreover, multivariate analysis suggested that CTSB expression might be an independent prognostic indicator for the survival of HCC patients after curative surgery.

**Conclusions:**

CTSB might be involved in the development and progression of HCC as an oncogene, and thereby may be a valuable prognostic marker for HCC patients.

**Electronic supplementary material:**

The online version of this article (doi:10.1186/s12943-016-0503-9) contains supplementary material, which is available to authorized users.

## Background

Hepatocellular carcinoma (HCC) grows rapidly and frequently associates with vascular invasion, metastasis, recurrence, and poor prognosis. HCC is the fifth most common human cancer and the third leading cause of cancer death worldwide [1, 2]. The increase in world-wide incidence and the high mortality of HCC highlight the importance of understanding the molecular mechanisms that trigger the neoplastic transformation of hepatocytes and the progression of HCC. However, the molecular mechanisms responsible for HCC development have not been well defined.

Proteases perform essential functions in such processes as ovulation [3], fertilization [4], bone remodeling [5], cell migration [6, 7], inflammation [8, 9], angiogenesis [10, 11], and apoptosis [12, 13]. CTSB, composed of a heavy chain of 25–26 kDa and a light chain of 5 kDa, belongs to a family of lysosomal cysteine proteases and plays an important role in intracellular proteolysis [14]. Numerous studies have shown that overexpression of CTSB is correlated with invasive and metastatic cancers. CTSB is known to interact with cystatins and annexin II tetramer (p11), which is also known as S100A10 [15]. These interactions place CTSB at crucial positions for the proteolytic activation of extracellular matrix (ECM) components, thereby enabling ECM degradation. It has been observed that the promoter of CTSB contains a GC-rich region including many SP1 sites, similar to a housekeeping gene [16]. These SP1 sites are known to increase in tumor cells [17]. Interestingly, it has been observed that CTSB is also involved in autophagy and cannibalism, as researchers have shown that tumor cannibalism is advantageous in tumor malignancy and is possibly involved in specific immune resistance, enabling tumor cells to recycle nutrients and maintain a proliferative and infiltrative phenotype [18]. This could also explain why the cores of highly infiltrative tumors are necrotic.

However, no research on CTSB has been done in HCC so far. To explore the exact role of CTSB in HCC, we investigated whether the expression of CTSB protein is different between tumor tissues and normal tissues, whether CTSB plays an important role in the development and progression of HCC, and whether CTSB is a prognostic factor in HCC after curative surgical treatment.

## Methods

### Patients and specimens

Fresh tumor tissue samples with paired non-cancerous liver tissue samples of 24 HCC patients were obtained in operation from the Nanfang hospital and Cancer Center of Southern medical university (SMUCC). A total of 168 paraffin-embedded HCC samples, which were histologically and clinically diagnosed in patients with radical surgery in NanFang hospital, between 2000 and 2007, were also included in this study. Resected specimens, fixed in 10 % formalin solution and then embedded in paraffin, were longitudinally sliced into 4-mm-thick sections. Representative sections were prepared and stained with hematoxylin and eosin for histologic examination. Western-blot was used to confirm the specificity of CTSB staining in fresh HCC tissues with paired non-cancerous liver tissues and cell lines MHCC-97H and MHCC-97 L. None of these patients had received radiotherapy or chemotherapy prior to surgical treatment. Clinical and pathological data of the 168 patients with HCC were collected, such as age, tumor size, stage, differentiation grade, and recurrence. The tumor stages were classified according to the 2010 TNM staging system of Union for International Cancer Control (UICC). Tumor differentiation was classified using the Edmondson grading system. Clinical follow-up information was obtained by telephone or from the outpatient records.

Written Ethics Approval and Patient Consent from the Research Ethics Committee of Nanfang Hospital and SMUCC were obtained. Participants were recruited and human experimentation was conducted in Nanfang Hospital and SMUCC. We have obtained written informed consent from all participants involved in the study.

### Cell culture

The HCC cell lines MHCC-97H, MHCC-97 L, Huh-7, HepG2, SMMC-7721, Bel-7404, and human hepatocyte cell line HL-7702 were obtained from The Cell Bank of Type Culture Collection of Chinese Academy of Sciences. MHCC-97H and MHCC-97 L were derived from the same parent cell MHCC-97 to ensure a similar genetic background and yet dramatic differences in spontaneous metastatic behavior. Compared with MHCC-97 L, which was not metastatic via subcutaneous inoculation, MHCC-97H featured more overt multidirectional metastasis. Cells were maintained in RPMI 1640 medium (Gibco, Invitrogen, Carlsbad, CA, USA) supplemented with 10 % fetal bovine serum (FBS; Hyclone, Logan, UT, USA), penicillin (100 units/ml), and streptomycin (100 units/ml) at 37 °C in humidified 5 % CO_2_ incubator.

### Western blot analysis

Equal amounts of protein lysates were separated by SDS-PAGE and transferred onto nitrocellulose membranes. Filters were probed with the following specific primary antibodies: anti-CTSB (Cell signaling, Danvers, MA, USA), MMP-9 (Abcam, Cambridge, MA, USA), β-actin (Sigma, St Louis, MO, USA), PI3K and phosphorylated-PI3K (Cell signaling, Danvers, MA, USA), Akt and phosphorylated-Akt (Cell signaling, Danvers, MA, USA), mTOR, and phosphorylated-mTOR (Cell signaling, Danvers, MA, USA). Blots were then incubated with horseradish peroxidase-conjugated secondary antibody (Pierce, Rockford, IL, USA) and visualized by chemiluminescence. The band density was quantified by densitometry using Scion Image software, and normalized to β-actin levels.

### Real-time RT-PCR analysis

Total RNA from human tissues was extracted using Trizol reagent (Invitrogen, Carlsbad, CA, USA) according to the manufacturer’s instructions. cDNA was synthesized from 1 μg of total RNA by use of the SuperScript® III First-Strand Synthesis System (Invitrogen, Carlsbad, CA). Real-time PCR was carried out using CFX96 Real-Time System (BIO-RAD). SYBR green 2× master mixture (Invitrogen, Carlsbad, CA, USA) was used in a total volume of 10 μL. The primer sequences were as follows: CTSB sense 5′- GGCCTCTATGACTCGCATGT -3′, antisense 5′- TTTGTAGGACGGGGTGTAGC -3′, GAPDH sense 5′-TGTTGCCATCAATGACCCCTT-3′, antisense 5′-CTCCACGACGTACTCAGCG-3′. GAPDH was used as an internal control. All reactions were run in triplicate in three independent experiments.

### Immunohistochemical analysis

Immunohistochemical (IHC) staining was performed using Dako Envision system (Dako, Carpinteria, CA) according to the manufacturer’s instructions. Briefly, 4 μm formalin-fixed paraffin-embedded sections were made and subsequently deparaffinized with xylenes, and rehydrated through graded ethanol series to distilled water. Antigen retrieval was done by heating sections with EDTA antigenic retrieval buffer (pH 8.0) in a microwave oven. Following endogenous peroxidase blocking in 0.3 % H_2_O_2_ for 15 minutes and nonspecific binding blocking with normal goat serum for 30 minutes, the sections were incubated with rabbit polyclonal anti-CTSB antibody (1:50; Santa Cruz Biotechnology) overnight at 4 °C. After sequential incubation with biotinylated anti-goat secondary antibody (Zymed) and streptavidin-horse-radish peroxidase (Zymed), reaction product was developed with Diaminobenzidine (DAB). Negative control was composed of mixture with no primary antibody but normal goat serum instead.

Three pathologists scored the immunohistochemically stained slides independently. Sum of intensity and extent score scaling from 0 to 7 was used as to assess and grade expression of CTSB into two groups: low (<3) and high (>3) expression.

### Vector construction and transfection

The pcDNA3.0 vector was used to generate pcDNA-CTSB. The CTSB shRNA Plasmid was purchased from Santa Cruz Biotechnology (Cat. no: sc-29238-SH). Vector transfection was performed according to the instructions. MHCC-97 L cell line was transfected with pcDNA expressing CTSB or empty vector, and MHCC-97H was used to knock-down the expression of CTSB. MHCC-97 L cells expressing CTSB or empty vector were selected for 14 days with G418 after infection. MHCC-97H cells transfected with CTSB-shRNA or Con-shRNA were selected for 14 days with puromycin after infection. In addition, MHCC-97H/CTSB-shRNA was transfected with pcDNA expressing MMP-9, and then used for the further experiments.

### Wound Healing Assay

Cell migration ability was assessed by measuring the movement of cells into scraped cellular area created by a 10 μl pipette tube when cells were grown to 80-90 % confluence in six-well culture plates. The phase contrast images of the wounds were recorded of 0, 24, 48 h and three separate experiments were performed. Cells transfected with empty vector and parental cells served as controls.

### Cell Invasion Assay

Upper chambers of 24-well transwell plate (Corning Incorporated, New York, NY, USA) were coated with 50 % Matrigel (BD Biosciences, Franklin, New Jersey, USA) in phosphate-buffered saline. Cells were incubated in the upper chamber. After 24 h incubation, invaded cells were stained with 0.5 % crystal violet, examined by bright field microscopy (OLYMPUS cx31, TOKYO, Japan), and photographed. Invasion rate was quantified by counting the invaded cells in five random fields per chamber under the fluorescence microscope (OLYMPUS IX71, TOKYO, Japan). Data summarized three independent experiments.

### Tumor formation in an animal model

Equivalent amounts of MHCC97L/CTSB cells and MHCC97H/CTSB-shRNA cells (5 × 10^5^ cells) were injected subcutaneously into the right flank of female BALB/c nude mice (Shanghai Slac Laboratory Animal Co. Ltd, Shanghai, China) at 5 weeks of age (15–17.5 g) respectively. Tumorigenesis procedure was observed by measuring solid tumors in 3 dimensions with a caliper for 21 days. Animals were sacrificed 21 days after injection. The experiments on mice had been approved by the ethics committee at SMUCC.

### Statistical analysis

Statistical analyses were performed using a statistical software package (SPSS19.0, Chicago, IL). The significance of CTSB mRNA levels was determined by *t*-test. The chi-square test was used to analyze the relationship between CTSB expression and clinicopathological characteristics. Survival times were evaluated using the Kaplan and Meier survival curves, and compared by the log-rank test. The significance of various variables for survival was analyzed by multivariate survival analysis using Cox’s regression model. P-value less than or equal to 5 percent was considered to be statistically significant.

## Results

### The Expression of CTSB in HCC Tissues and cell lines

**Fig. 1:**
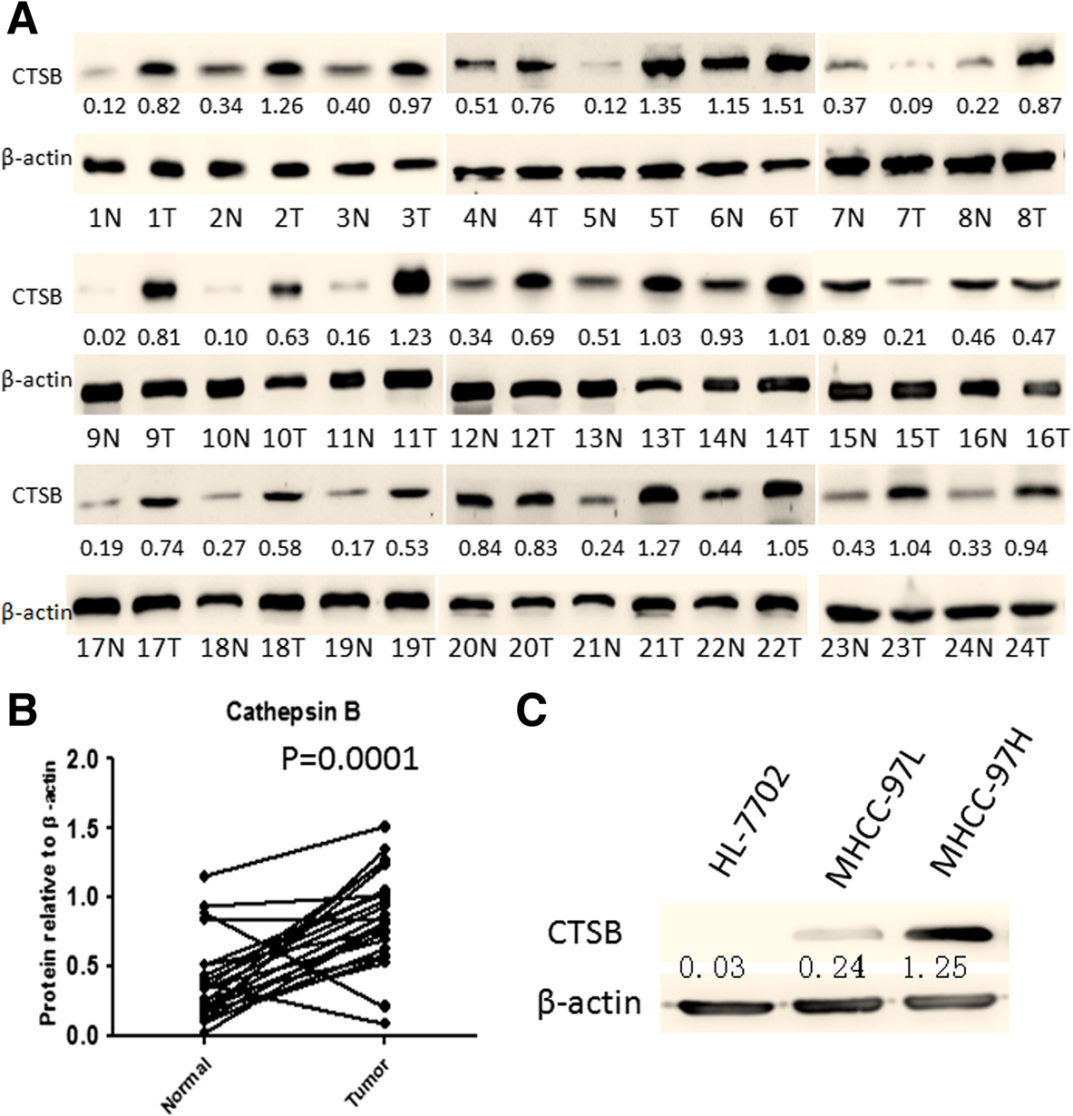
Expression levels of CTSB in HCC tissues as well as HCC cell lines. **a** Expression levels of CTSB protein in 24 paired HCC tissues by Western blotting. N, paracarcinoma (normal) liver tissues. T, HCC tissues. **b** Quantitative analysis of CTSB protein in 24 paired HCC tissues. **c**. Western blot analysis of CTSB protein expression in one hepatocyte cell line (HL-7702) and two HCC cell lines (MHCC-97 L and MHCC-97H)

To determine the expression of CTSB protein in HCC tissues, Western blotting was performed in 24 HCC tissues with paired non-cancerous tissues. Among 22 of 24 HCC tissues with paired normal tissues, clearly increased levels of CTSB expression were detected in all the tumors tissues in comparison to the paired non-cancerous tissues (Fig. 1a and b). However, the level of CTSB expression was higher in non-cancerous tissues than that in tumors tissues in the rest of 2 paired of HCC tissues (Fig. 1a, patient samples No. 7 and No. 15). In addition, we first examined the expression of CTSB in HL-7702, MHCC-97 L, and MHCC-97H to investigate whether there was any correlation between CTSB expression and the metastasis potential in HCC. As presented in Fig. 1c, the level of CTSB protein in MHCC-97H was much higher than that in MHCC-97 L and HL-7702. No statistically significant difference was found between MHCC-97 L and HL-7702. The RT-PCR analysis had a result similar to that of Western blot (data not shown). As expected, the highly metastatic cell line (MHCC-97H) exhibited stronger signals of CTSB than the lowly metastatic cell line (MHCC-97 L). In HL-7702cells, CTSB was almost undetectable. These data suggested that CTSB might serve as an oncogene in HCC.

**Fig. 2:**
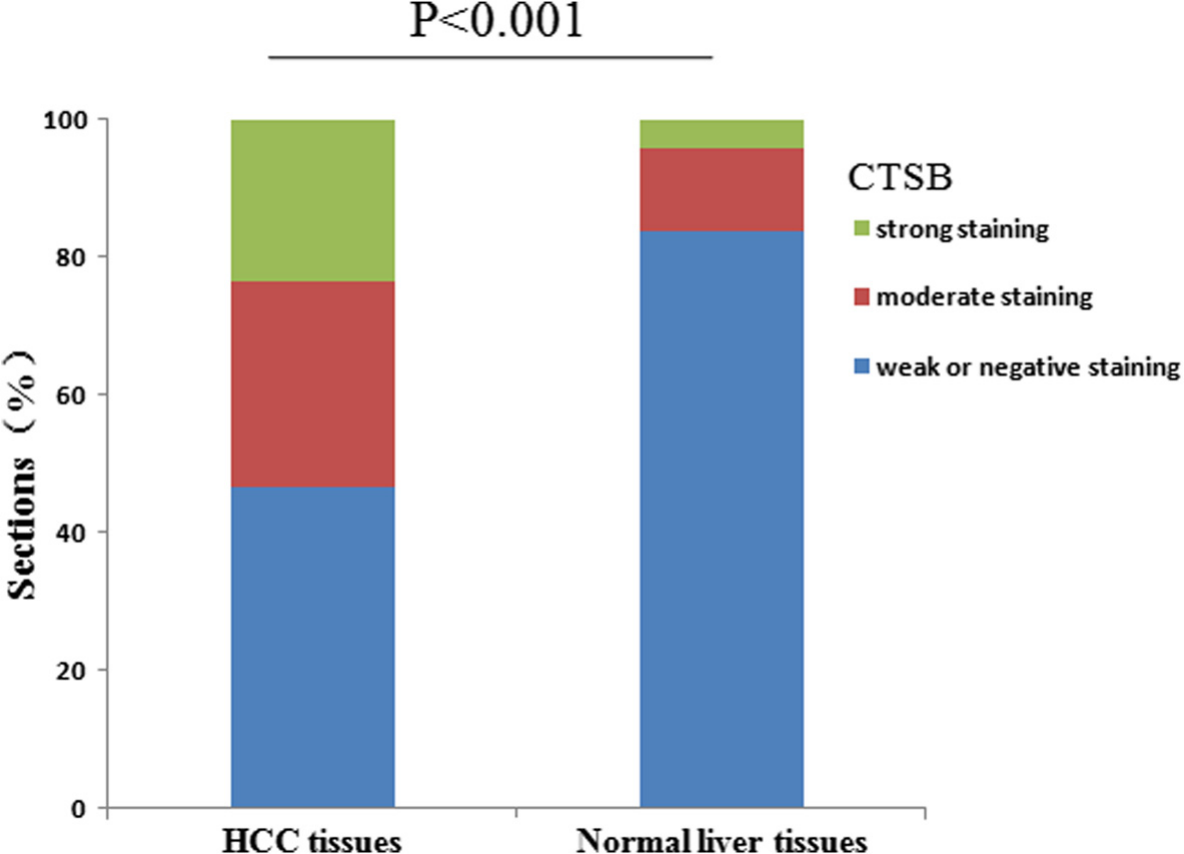
Analysis of CTSB protein in tissues by immunohistochemistry. CTSB expression was significantly increased in HCC tissues, when compared with normal liver tissues, *P* < 0.001

To verify this observation, we further examined the expression of CTSB protein in 168 paraffin-embedded HCC samples and 37 normal liver (non-cancerous) samples by immunohistochemical analysis. As shown by immunohistochemical analysis, 78 of 168 (46.5 %) paraffin-embedded HCC tissues showed weak or negative staining of CTSB protein, while 50 of 168 (29.8 %) HCC tissues showed mainly moderate CTSB staining (in the membrane and cytoplasm of cancer cell), and 40 of 168 (23.7 %) showed strong staining in tumor cells. 31 of the 37 non-cancerous tissues indicated negative staining of CTSB and the rest of six non-cancerous tissues showed weak expression (Fig. 2).

Additionally, the incidence of CTSB protein expression in well-differentiated carcinoma was significantly lower than that in poorly differentiated tumors, and CTSB expression was significantly related with tumor differentiation (*P* = 0.003) (Table 1).

**Table 1:** Relationship between CTSB expression and clinicopathologic features of HCC patients

Clinicopathologic parameters	n	CTSB expression		*P* value
		Positive (%)	Negative (%)	
All cases	168	90 (53.5 %)	78 (46.5 %)	
Gender				0.562
Male	110	57 (51.8 %)	53 (48.2 %)	
Female	58	32 (55.2 %)	26 (44.8 %)	
Age (years)				0.310
<50	123	61 (49.6 %)	62 (50.4 %)	
≥50	45	26 (57.8 %)	19 (42.2 %)	
Tumor size (cm)^a^				0.622
<5	131	71 (54.2 %)	60 (45.8 %)	
≥5	37	19 (51.4 %)	18 (48.6 %)	
Serum HBsAg				0.125
Positive	147	82 (55.8 %)	65 (44.2 %)	
Negative	21	10 (47.6 %)	11 (52.4 %)	
Serum AFP (ng/ml)				0.814
<25	50	25 (50.0 %)	25 (50.0 %)	
≥25	118	60 (50.8 %)	58 (49.2 %)	
Cirrhosis				0.078
Presence	145	78 (53.8 %)	67 (46.2 %)	
Absence	23	10 (43.5 %)	13 (56.5 %)	
UICC stage				0.004
I + II	100	32 (32.0 %)	68 (68.0 %)	
III + IV	68	48 (70.6 %)	20 (29.4 %)	
Metastasis/Recurrence				0.000
Yes	133	88 (66.2 %)	45 (33.8 %)	
No	35	12 (34.3 %)	23 (65.7 %)	
Edmondson grade				0.003
Low (I/II)	82	32 (39.0 %)	50 (61.0 %)	
High (III/IV)	86	56 (65.1 %)	30 (34.9 %)	

^a^:The largest dimension of the tumor specimen

### Correlation of CTSB expression with clinicopathological features and Outcomes

The association between CTSB expression and the clinicopathological outcomes is shown in Table 1. CTSB expression was significantly correlated with stage, recurrence, and tumor differentiation. There was no significant correlation between CTSB expression and age, gender, tumor size, serum HBsAg, serum AFP, or liver cirrhosis (Table 1).

### Correlation of CTSB expression with overall survival

**Fig. 3:**
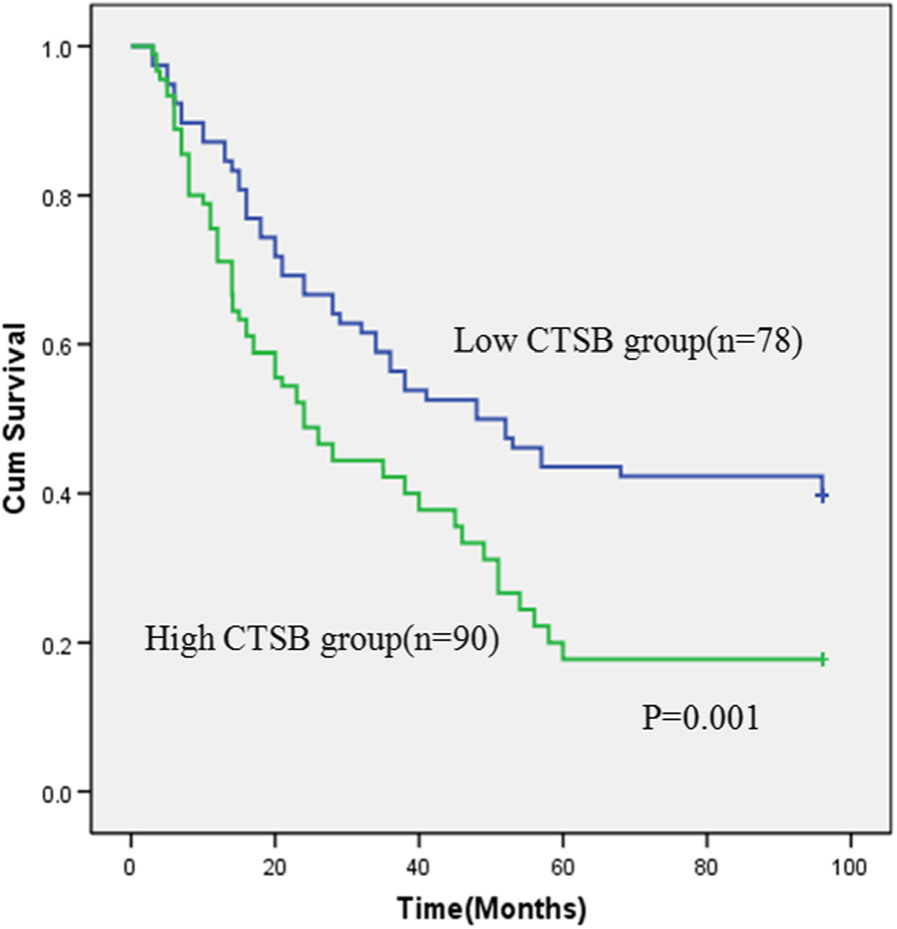
Survival curves for patients with high CTSB expression versus low CTSB-expressing carcinoma. The 5-year overall survival rate was 19.3 % in the high CTSB protein expression group (green line), but it was 43.4 % in the low expression group (blue line), *P* = 0.032

The median follow-up time for overall survival was 72 months for all patients. The 2-year and 5-year overall rates for all patients were 53.4 % and 29.7 %, respectively. Among these patients, the overall survival of the patients with low CTSB expression (5-year overall rate, 43.4 %) was significantly higher than the high CTSB expression group (5-year overall rate, 19.3 %) (*P* =0.001, Fig. 3). Besides CTSB expression level, tumor size, serum AFP, liver cirrhosis, stage, tumor recurrence, and tumor differentiation were also significantly correlated with overall survival in univariate analysis (Table 2). Furthermore, overall survival was possibly correlated with age (*P* =0.053).

**Table 2:** Univariate survival analysis of 168 patients with HCC

Variable	n	Overall survival		*P* value
		2-year	5-year	
CTSB expression				0.001
Positive	90	42.5 %	19.3 %	
Negative	78	62.8 %	43.4 %	
Gender				0.518
Male	110	57.9 %	33.4 %	
Female	58	60.3 %	39.6 %	
Age (years)				0.053
<50	123	61.2 %	40.9 %	
≥50	45	54.1 %	35.4 %	
Tumor size (cm)^a^				0.001
<5	131	68.2 %	47.6 %	
≥5	37	49.8 %	27.2 %	
Serum HBsAg				0.088
Positive	147	54.3 %	36.4 %	
Negative	21	62.3 %	43.1 %	
Serum AFP (ng/ml)				0.001
<25	50	70.3 %	51.3 %	
≥25	118	52.6 %	33.3 %	
Cirrhosis				0.031
Presence	145	52.9 %	23.3 %	
Absence	23	63.7 %	43.6 %	
UICC stage				0.001
I + II	100	75.2 %	49.8 %	
III + IV	68	50.1 %	24.5 %	
Metastasis/Recurrence				0.000
Yes	133	43.4 %	23.3 %	
No	35	67.2 %	40.7 %	
Edmondson grade				0.005
Low (I/II)	82	66.9 %	42.8 %	
High (III/IV)	86	48.3 %	27.1 %	

^a^:The largest dimension of the tumor specimen

The Cox proportional hazards mode was employed to evaluate the effects of the independent factors on overall survival. These factors include CTSB expression, gender, age, tumor size, Serum HBsAg, serum AFP, tumor size, liver cirrhosis, stage, tumor recurrence, and tumor differentiation. The results showed that CTSB expression, serum AFP, tumor size, tumor recurrence and stage were recognized as independent prognostic factors of survival (Table 3). Therefore, Multivariate analysis indicated that CTSB protein expression has a significant correlation with poor prognosis of HCC patients as an independent factor.

**Table 3:** Cox regression analysis of patients with HCC

Variables	Univariate		*P* value
	HR	CI (95 %)	
CTSB expression (1 = down,2 = over)	5.132	3.125 ~ 6.037	0.000
Gender (1 = male,2 = female)	0.511	0.327 ~ 1.078	0.106
Age (1 < 50,2 ≥ 50)	0.468	0.279 ~ 0.998	0.412
Serum HBsAg (1 = negative,2 = positive)	1.033	0.723 ~ 2.411	0.107
Serum AFP (1 < 25 ng/ml,2 ≥ 25 ng/ml)	3.415	2.379 ~ 4.866	0.009
Tumor size (1 < 5 cm,2 ≥ 5 cm)	1.756	1.218 ~ 3.321	0.039
Cirrhosis (1 = Absence,2 = Presence)	0.932	0.782 ~ 1.687	0.443
Metastasis/Recurrence (1 = no,2 = yes)	5.002	3.437 ~ 7.312	0.000
UICC stage (1 = I + II,2 = III + IV)	1.958	1.021 ~ 4.190	0.042
Edmondson grade (1 = High (III/IV),2 = Low (I/II))	1.412	0.783 ~ 2.224	0.098

### Alteration of CTSB expression regulated migration and invasion capabilities of HCC cells In Vitro

**Fig. 4:**
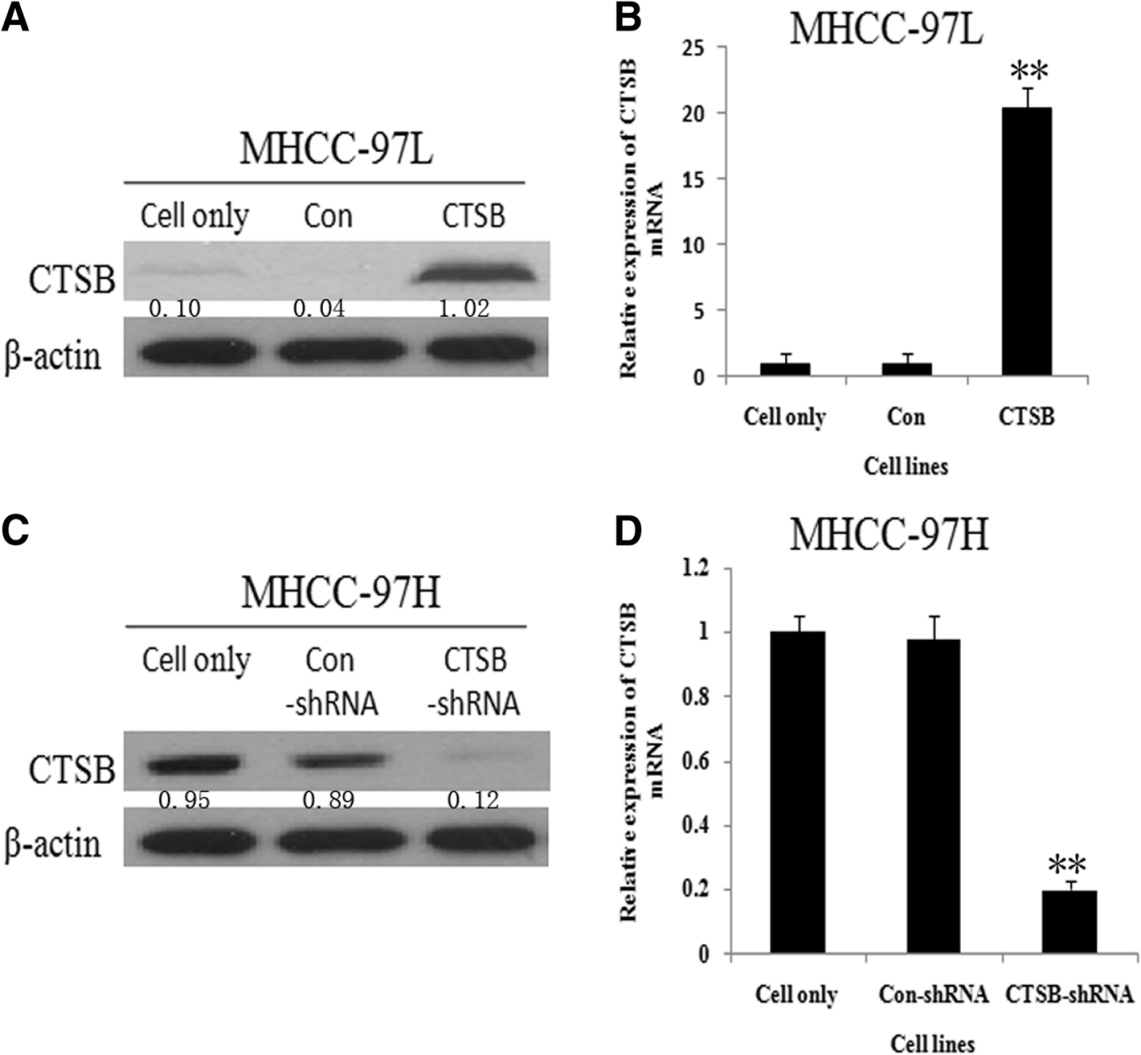
CTSB expression in MHCC-97 L and MHCC-97H. **a** CTSB Protein of MHCC-97 L cells stably transfected with pcDNA-CTSB increased most significant compared to MHCC-97 L/Con cells and MHCC-97 L cells. As detected by Western blotting. **b** MHCC-97 L/CTSB also show the highest CTSB mRNA among the three. As detected by RT-PCR. **c** CTSB specific shRNA resulted in the reduction of CTSB protein in MHCC-97H cells. **d** MHCC-97H/CTSB-shRNA show the lowest CTSB mRNA among the three. The expression of β-actin mRNA and protein was also examined and served as control for sample loading. (**P < 0.01 as compared to parental groups, *P < 0.05 as compared to parental groups)

**Fig. 5:**
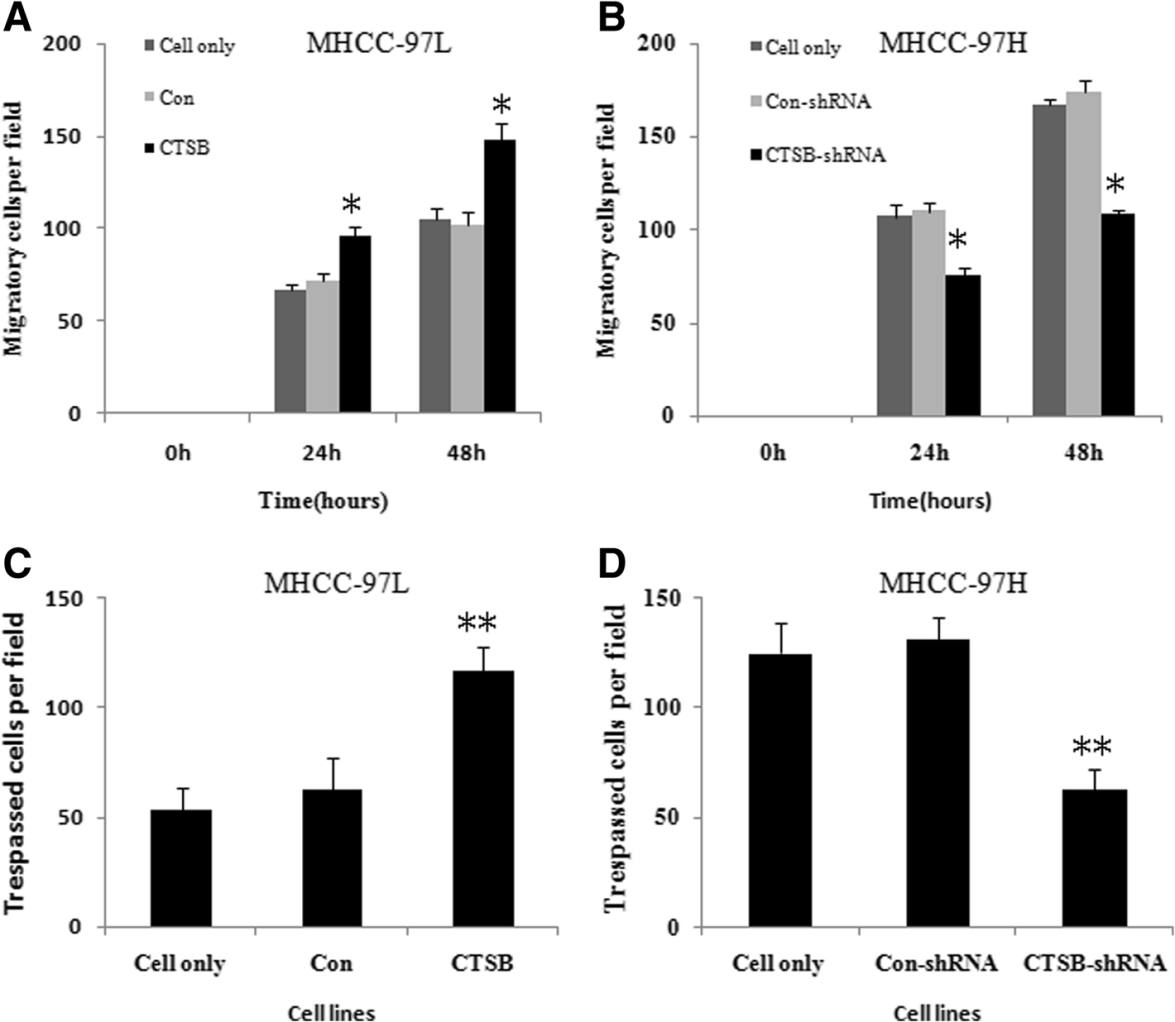
Effect of CTSB on HCC cell migration and invasion. **a** Effect of overexpression of CTSB on the cell migration of MHCC-97 L cells. As detected by Wounding-healing assay. Migratory cells were quantified by counting three separate fields in different wells. The migratory ability of MHCC-97 L/CTSB cells was remarkably increased compared to that of MHCC-97 L/Con and MHCC-97 L cells. **b** Effect of CTSB down-regulation on the cell migration of MHCC-97H cells. MHCC-97H/CTSB-shRNA exhibited reduced cell motility compared with control. **c** Effect of overexpression of CTSB on the invasive potential of MHCC-97 L cells. The number of cells that pass through Matrigel in MHCC-97 L/CTSB group was higher than that in control groups. As detected by transwell assay. **d** Effect of CTSB down-regulation on the invasive potential of MHCC-97H cells. The number of cells that pass through Matrigel in MHCC-97H/CTSB-shRNA group was lower than that in control groups. All the results were represented as the mean ± SD from three independent experiments. (**P < 0.01 as compared to parental groups, *P < 0.05 as compared to parental groups)

We successfully constructed stable clones over-expressing CTSB from MHCC-97 L cells (Fig. 4a and b). We employed wound healing assay to detect the effect of CTSB on cell migration. As shown in Fig. 5a, an evident acceleration in the wound closure rate was observed in MHCC-97 L/CTSB cells at 48 h compared with the controls (*P* < 0.05). In the transwell assay (Fig. 5c), the number of cells that passed through Matrigel in MHCC-97 L/CTSB group was higher than that in the control groups (*P* < 0.05). As expected, overexpression of CTSB was accompanied by the enhanced invasiveness of MHCC-97 L cells.

The endogenous CTSB level in MHCC-97H was stably knocked down by transfecting small interfering RNA (Fig. 4c and d). Contrary to the effect of overexpression of CTSB, down-regulation of endogenous CTSB in MHCC-97H cells suppressed the migration and invasion abilities compared with their controls (Fig. 5b and d).

Protein level of CTSB of four HCC cell lines was shown in Additional file 1: Figure S1A. Huh-7 and Bel-7404 expressed the lowest and highest levels of CTSB among four HCC cell lines respectively. Thus, huh-7 cells were transfected with pcDNA expressing CTSB, and Bel-7404 cells were used to knock down CTSB expression. We successfully established stable Huh-7 clones over-expressing CTSB (Additional file 1: Figure S1B). In the transwell assay (Additional file 1: Figure S1C), Huh-7/CTSB displayed remarkable enhanced invasive ability as compared with either Huh-7/Con or Huh-7. In addition, Bel-7404 cells transfected with CTSB-shRNA had significantly reduced level of CTSB protein than with Con-shRNA, respectively (Additional file 1: Figure S1D). We also employed transwell assay to explore the effect of CTSB on cell invasion. As shown in Additional file 1: Figure S1E, Bel-7404/CTSB-shRNA showed much weaker invasive activity than either Bel-7404 or Bel-7404/Con-shRNA.

### CTSB might regulate MMP-9 through PI3K/Akt signaling pathway

**Fig. 6:**
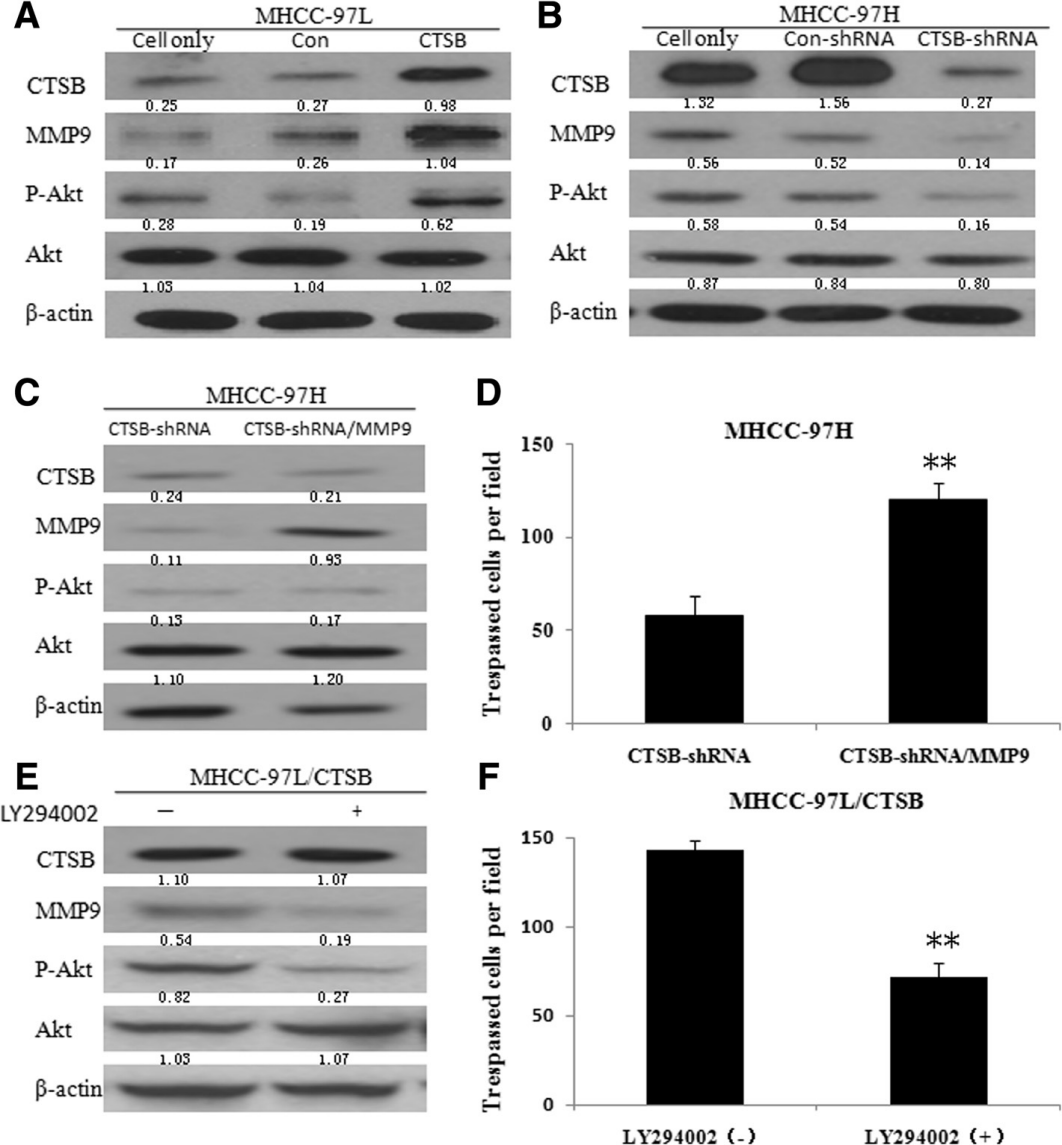
CTSB regulates MMP-9 through PI3K/Akt signal pathway. **a** Stable expression of CTSB in MHCC-97 L increased the protein level of p-Akt and MMP-9. **b** Down-regulation of CTSB in MHCC-97H decreased the protein level of p-Akt and MMP-9. **c** MMP-9 protein of MHCC-97H/CTSB-shRNA cells stably transfected with pcDNA-MMP-9 increased most significant compared to MHCC-97H/CTSB-shRNA cells. **d** CTSB knockdown cells stably transfected with MMP-9 showed increased invasive ability. **e** Treatment of MHCC-97 L/CTSB cells with LY294002, a PI3K inhibitor, led to down-regulation of MMP-9 at protein levels. **f** MHCC-97 L/CTSB cells treated with LY294002 show decreased invasive ability. (**P < 0.01 as compared to parental groups, *P < 0.05 as compared to parental groups)

MMP-9 has been implicated in HCC invasion and metastasis, and up-regulation of MMP-9 can be achieved by activation of PI3K/Akt pathways [19]. Western blot analysis showed that the expression of p-Akt was significantly increased in CTSB-overexpressing cells (Fig. 6a), indicating that up-regulation of CTSB is able to enhance Akt signaling, which in turn leads to concomitant up-regulation of MMP-9 expression. In addition, inhibition of CTSB resulted in significant diminishments of p-Akt and MMP-9 expression in MHCC-97H cells (Fig. 6b). Furthermore, upregulation of MMP-9 partially rescued the ability of CTSB knockdown cells to invade the Matrigel-coatedmembrane (Fig. 6c and d), indicating that CTSB is a major mediator of MMP-9 induction. This notion was further supported by the results that MMP-9 expression was decreased but CTSB expression was unchanged in MHCC97L/CTSB cells after treatment with the PI3K/Akt inhibitorLY294002 (Fig. 6e), which led to decreased invasive ability of CTSB-overexpressing cells (Fig. 6f).

In addition, as shown in Additional file 2: Figure S2, Western blot was applied to detect the expression of components of PI3K transduction in HCC cell lines. Protein levels of p-PI3K, p-Akt, and p-mTOR in MHCC-97H/CTSB-shRNA were lower than both control groups (MHCC-97H and MHCC-97H/Con-shRNA), while in MHCC-97 L/CTSB were higher than both control groups (MHCC-97 L and MHCC-97 L/Con). The findings above suggested that the effects of CTSB in HCC cells are partially mediated by PI3K/Akt signaling.

### CTSB enhanced Tumorigenicity in vivo

**Fig. 7:**
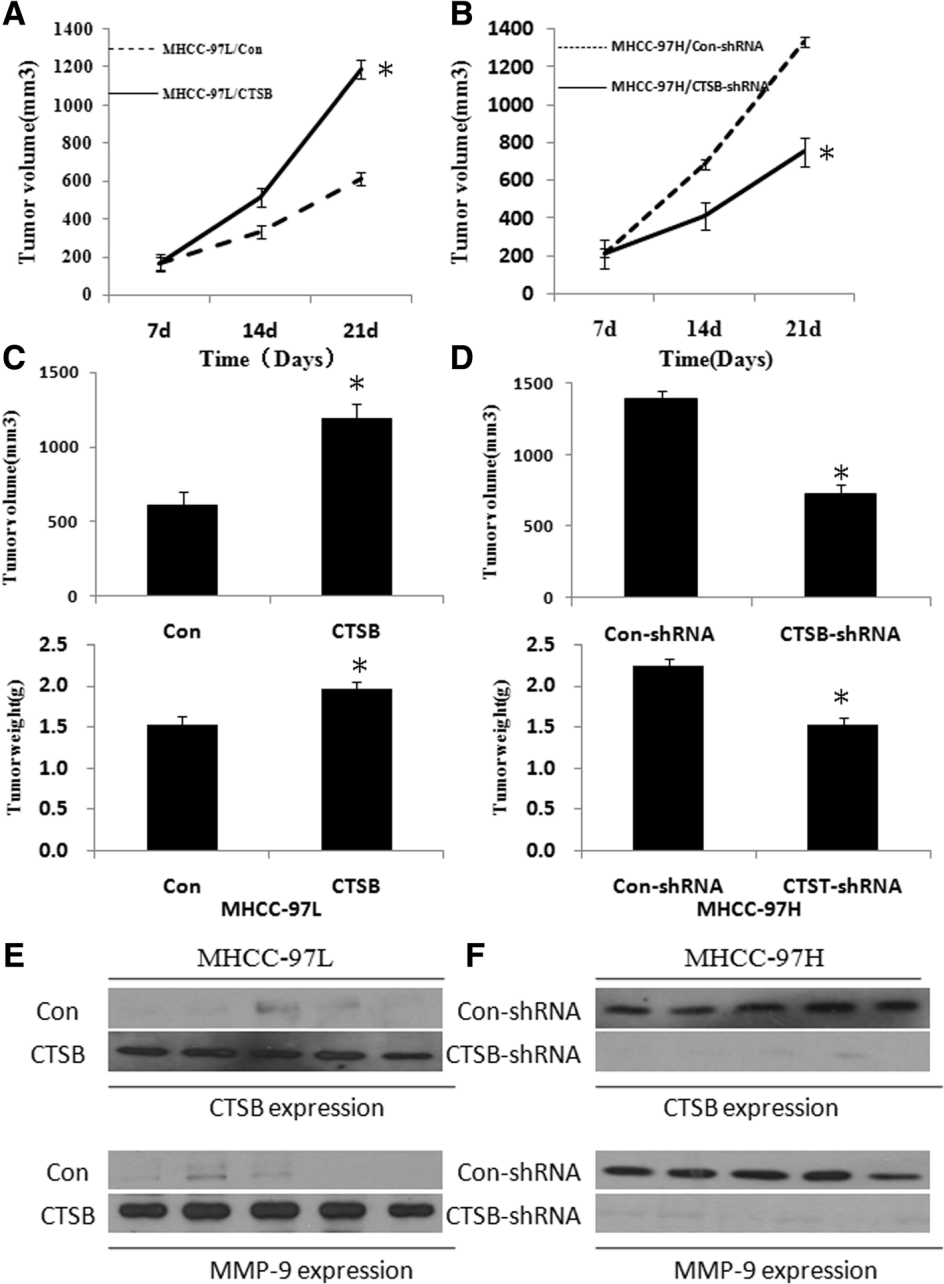
Effect of CTSB on subcutaneous tumorigenicity of MHCC-97 L and MHCC-97H. **a** Tumor growth curve of after injection of nude mice with CTSB or control vector expressing MHCC-97 L cells. **b** Tumor growth curve of nude mice injected by MHCC-97H cells transfected with CTSB-shRNA or Con-shRNA. **c** MHCC-97 L/CTSB tumors showed a significant increase in tumor volume and weight compared with control tumors on day 21 after injection. **d** MHCC-97H/CTSB-shRNA tumors showed a significant decrease in tumor volume and weight compared with control tumors on day 21 after injection. **e** Western blot analysis of the expression of CTSB in MHCC-97 L/CTSB tumors and control tumors. **f** Western blot analysis of the expression of CTSB in MHCC-97H/CTSB-shRNA tumors and control tumors

To further investigate the role of CTSB in HCC progression, we assessed the effect of CTSB up-regulation on the growth of HCC xenograft tumors in nude mice injected with CTSB-overexpressing cells or control cells. MHCC-97 L/CTSB tumors grew much faster than control tumors (Fig. 7a). At the end of the study day 21, tumor weight of the control group (1.30 ± 0.07 g) was only 75 % of the MHCC-97 L/CTSB group (1.96 ± 0.06 g) (Fig. 7c). To correlate the biological response with the mechanisms identified in cells, MMP-9 protein was assessed by Western blot analysis. As shown in Fig. 7e, MMP-9 expression was significantly increased in five representative MHCC-97 L/CTSB tumors. In addition, down-regulation of CTSB showed the opposite effects (Fig. 7b, d, and f). The results indicate that CTSB is able to promote HCC tumor growth in nude mice.

## Discussion

The long-term survival of HCC patients remains unsatisfactory because of its high incidence of recurrence and metastasis after hepatic resection. Thus, identification of effective biomarkers for HCC patients is certainly a great concern of medical researchers.

As an intense interest of current research, CTSB has been proposed as a potentially effective biomarker for a variety of cancers [20–25]. Many researchers concluded that CTSB might be a metastasis-related gene, and consequently in the clinic, it can serve as a predictor for therapeutic outcome or prognosis of patients with cancers [26–34]. In this study, we found that 23.4 % paraffin-embedded HCC cancer tissues showed strong membrane and cytoplasm staining of CTSB, 40.2 % HCC tissues showed moderate CTSB staining, and 36.4 % showed negative staining in tumor cells, while the non-cancerous tissues presented mainly negative expression of CTSB, indicating that CTSB might play an important role in the development and progression of HCC. Furthermore, as determined by immunohistochemistry, the incidence of CTSB protein expression in poorly differentiated carcinomas was significantly higher than that in well-differentiated tumors, suggesting that high level of CTSB expression was related to poor tumor differentiation. Additionally, we have shown that CTSB expression was correlated with stage, recurrence, and tumor differentiation. There was no significant correlation between CTSB expression and age, gender, Tumor size, Serum HBsAg, Serum AFP, or liver cirrhosis. Our study suggests that high level of CTSB expression might be positively correlated with worse tumor biological features, such as rapid tumor progression and metastases. Furthermore, by univariate analysis of the Cox proportional-hazard model, CTSB expression, serum AFP, tumor size, metastasis/recurrence, and TNM staging were found to be associated with an increased risk of death by HCC. Thus, CTSB might be a powerful prognostic index of survival in HCC.

To confirm the findings above, we compared CTSB expression in two HCC cell lines with different metastatic potential. As shown in the results section, the cell line with high metastatic potential expressed more CTSB mRNA and protein than did the cell line with low metastatic potential. This also means that HCC cells with higher metastatic potential contain more CTSB. CTSB is considered to play various roles in regulating cellular functions depending on specific cell type, substratum and other factors. It has been shown to increase migration and invasion of fibroblast-like synoviocytes, to promote migration of glioma-initiating cells, and to induce apoptosis of porcine oocyte. Although many studies have been performed to determine the functional features of CTSB in different tumors, little attention has been paid to regulation of CTSB in HCC. To further testify the role of CSTB in HCC, a series of relevant functional experiments, from positive to negative, as well as from in vitro to in vivo, were performed in our study.

A previous study indicated that the two cell lines derived from the same host cell line, MHCC-97H and MHCC-97 L, could provide an ideal cell model system for the study of HCC metastasis and proliferation. To understand the functions of CTSB, we established two target cell lines: MHCC-97H silencing CTSB and MHCC-97 L overexpressing CTSB. We observed an association of up-regulation of CTSB with promotion of cell migration and invasion in vitro, as well as an association of knockdown of CTSB with the reverse. Additionally, our study has also provided the first validation about the oncogenic capacity of CTSB expression in vivo. Up-regulation of CTSB in MHCC-97 L cells led to an enhancement of tumorigenicity potential in mice, while down-regulation of CTSB in MHCC-97H cells was correlated with inhibition of tumoregenicity. All the results of the functional experiments are in agreement with the findings derived from our clinical data. By demonstrating the potential role of CTSB in HCC cell lines, we hope that our study will provide a new insight into CTSB in HCC progression.

Invasion is a characteristic feature of HCC and a major poor prognostic factor in patients with HCC. Invasion of malignant HCC requires degradation of the extracellular matrix and the basement membrane. MMPs play an important role in the proteolytic destruction of extracellular matrix and basement membranes, they are therefore essential for tumor invasion [35]. MMP-9 has also been implicated in HCC invasion and metastasis, and upregulation of MMP-9 can be achieved by activation of PI3K/Akt pathways [19]. In this study, we have shown that CTSB is able to enhance PI3K/Aktoncogenic pathways in HCC cells. Therefore, CTSB may enhance cell invasiveness by promoting MMP-9 expression both in vitro and in vivo via PI3K/Akt pathways. Furthermore, we also found that overexpression of stably transfected MMP-9 partially rescued the invasiveness of CTSB knockdown cells. In addition, previous study showed that CTSB could enhance the activity of the matrix metalloproteinases (MMPs) by destroying their inhibitors (e.g., TIMP1 and TIMP2) in human articular chondrocytes and maintain a high level of MMPs, thereby promoting ECM degradation [36]. The above results suggested that MMP-9 is a major downstream effector of CTSB in regulating cell migration and invasion.

CTSB expression status, combined with clinicopathological features and other biomarkers of HCC, may be useful to stratify patients for individual treatment, such as those of targeted therapy or chemotherapy. Since the number of the cases in our study was not too big, the relationship between CTSB expression and metastases still requires to be evaluated. Therefore, further studies are needed to clarify the mechanisms by which CTSB is involved in the development and progression of HCC.

## Conclusions

In summary, CTSB was up-regulated in HCC. The expression level of CTSB was correlated with poor prognosis in patients with HCC. In addition, our study also provides a better understanding in both the molecular mechanism and functional role of CTSB in human HCC. Our current work revealed that CTSB has an oncogenic role in hepatocarcinogenesis by facilitating cancer cell migration and invasion. Notably, CTSB can up-regulate MMP-9 expression through PI3K/Akt signaling in HCC. Our study indicated that CTSB was an independent risk factor for poor prognosis and might be a useful biomarker for therapeutic strategy and control in HCC treatment.

## Additional files

Additional file 1: Figure S1. Expression of CTSB in HCC cell lines. (A) CTSB protein expression levels in MHCC-97H, MHCC-97 L, Huh-7, HepG2, SMMC-7721, and Bel-7404 cell lines were determined by Western blot. (B) CTSB Protein of Huh-7 cells stably transfected with pcDNA-CTSB increased most significant compared to Huh-7/Con cells and Huh-7 cells. (C) Effect of overexpression of CTSB on the invasive potential of Huh-7 cells. The number of cells that passed through Matrigel in Huh-7/CTSB group was higher than that in control groups. (D) CTSB specific shRNA resulted in the reduction of CTSB protein in Bel-7404 cells. (E) Effect of CTSB down-regulation on the invasive potential of MHCC-97H cells. The number of cells that passed through Matrigel in Bel-7404/CTSB-shRNA group was lower than that in control groups. (***P* < 0.01 as compared to parental groups, **P* < 0.05 as compared to parental groups). (TIF 538 kb) Additional file 2: Figure S2. Effect of CTSB on the PI3K pathway. (A) Down-regulation of CTSB decreased the protein levels of p-PI3K, p-Akt, and p-mTOR. (B) Stable expression of CTSB increased the protein levels of p-PI3K, p-Akt, and p-mTOR (TIF 408 kb)

## Abbreviations

CTSB: cathepsin B
HCC: hepatocellular carcinoma
IHC: immunohistochemical
SMUCC: cancer center of Southern medical university
UICC: union for international cancer control
