# Supplementary figures and images for: Over-expression of cathepsin B in hepatocellular carcinomas predicts poor prognosis of HCC patients
Source: Mol Cancer. 2016 Feb 20;15:17. doi: 10.1186/s12943-016-0503-9 (PMC4761221; doi:10.1186/s12943-016-0503-9)

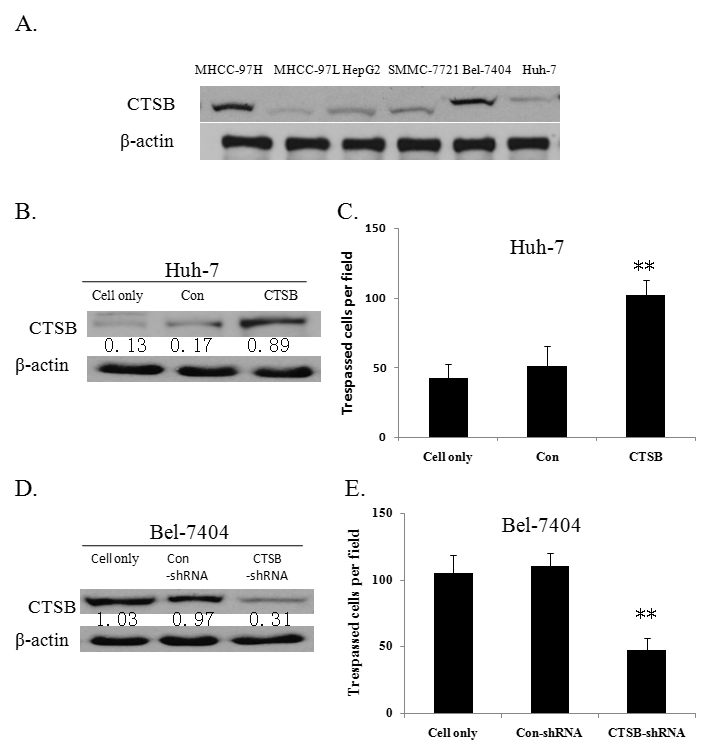

Supplement: Additional file 1: Figure S1. — Expression of CTSB in HCC cell lines. (A) CTSB protein expression levels in MHCC-97H, MHCC-97 L, Huh-7, HepG2, SMMC-7721, and Bel-7404 cell lines were determined by Western blot. (B) CTSB Protein of Huh-7 cells stably transfected with pcDNA-CTSB increased most significant compared to Huh-7/Con cells and Huh-7 cells. (C) Effect of overexpression of CTSB on the invasive potential of Huh-7 cells. The number of cells that passed through Matrigel in Huh-7/CTSB group was higher than that in control groups. (D) CTSB specific shRNA resulted in the reduction of CTSB protein in Bel-7404 cells. (E) Effect of CTSB down-regulation on the invasive potential of MHCC-97H cells. The number of cells that passed through Matrigel in Bel-7404/CTSB-shRNA group was lower than that in control groups. (**P < 0.01 as compared to parental groups, *P < 0.05 as compared to parental groups). (TIF 538 kb) [file 12943_2016_503_MOESM1_ESM.tif]

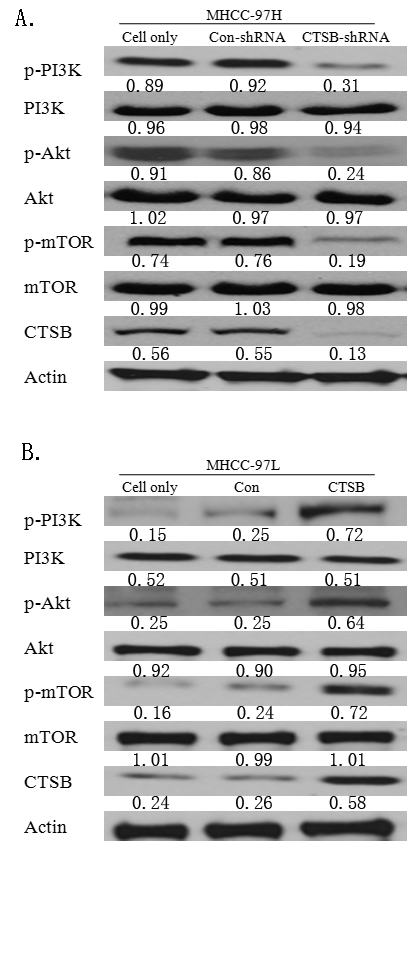

Supplement: Additional file 2: Figure S2. — Effect of CTSB on the PI3K pathway. (A) Down-regulation of CTSB decreased the protein levels of p-PI3K, p-Akt, and p-mTOR. (B) Stable expression of CTSB increased the protein levels of p-PI3K, p-Akt, and p-mTOR (TIF 408 kb) [file 12943_2016_503_MOESM2_ESM.tif]
